# A Machine Learning-Assisted Recognition and Compensation Method for UWB Ranging Errors in Complex Indoor Environments

**DOI:** 10.3390/s26082434

**Published:** 2026-04-15

**Authors:** Jiayuan Zhang, Guangxu Zhang, Ying Xu, Zeyu Li, Hao Wu

**Affiliations:** College of Geodesy and Geomatics, Shandong University of Science and Technology, Qingdao 266590, China; 202483020204@sdust.edu.cn (J.Z.); 202382020073@sdust.edu.cn (G.Z.); lizeyu@sdust.edu.cn (Z.L.); 202383020042@sdust.edu.cn (H.W.)

**Keywords:** machine learning, indoor positioning, non-line-of-sight (NLOS), ranging error mitigation, ultra-wideband (UWB)

## Abstract

**Highlights:**

**What are the main findings?**

- A lightweight NLOS recognition pipeline using a two-dimensional UWB feature vector composed of the ranging value and the power difference (TR−FP) achieves stable multi-class occlusion identification (LOS/door/wall/body) in complex indoor environments.
- A sliding-window threshold consistency check effectively suppresses frame-level label jitter, leading to more reliable environment labels for downstream correction.
- Environment-specific MLP compensation models significantly reduce ranging bias and improve positioning accuracy when integrated with nonlinear least squares (NLS) localization.

**What are the implications of the main findings?**

- The proposed framework can be deployed on low-cost UWB modules without raw CIR parsing, reducing implementation burden while maintaining robust performance under multipath and dynamic occlusions.
- By coupling temporally consistent recognition with environment-adaptive correction, the method offers a practical route to enhance indoor UWB reliability for applications such as industrial tracking, smart buildings, and emergency response.

**Abstract:**

Ultra-wideband (UWB) technology has been widely adopted for indoor positioning due to its high temporal resolution. However, the accuracy of UWB-based indoor positioning is fundamentally limited by ranging measurement errors, particularly under non-line-of-sight (NLOS) conditions, where systematic bias and uncertainty are introduced into the measured distances. In this paper, a measurement error mitigation method is proposed to improve UWB ranging reliability in complex indoor environments. The method first identifies NLOS measurements using low-dimensional physical features and a lightweight machine learning classifier. Subsequently, an error compensation strategy is applied to correct biased ranging observations, which are then incorporated into a nonlinear least squares positioning model. Experimental results obtained in typical indoor environments demonstrate that the proposed method significantly reduces ranging errors and improves positioning accuracy compared with conventional approaches. The results indicate that the proposed framework effectively enhances measurement robustness without increasing system complexity.

## 1. Introduction

In recent years, with the rise of smart cities and the Internet of Things (IoT) technologies, Location-Based Services (LBSs) have developed significantly. LBS applications mainly cover outdoor and indoor scenarios. In open outdoor environments, the Global Navigation Satellite System (GNSS) has been able to provide high-precision positioning services [1]. However, GNSS performance degrades significantly in indoor and dense urban environments due to signal blockage, multipath interference, and limited penetration capability [2]. Therefore, reliable indoor positioning technologies have become an important research focus. Indoor positioning technologies can generally be divided into two categories [3]: one based on radio frequency (RF) signals, such as Ultra-Wideband (UWB) positioning [4], WiFi positioning [5], Bluetooth positioning [6], etc.; the other is based on sensors built into mobile terminals, such as Pedestrian Dead Reckoning (PDR) [7], light source positioning [8], sound source positioning [9], and visual positioning [10]. Different positioning technologies have different application scenarios. Among them, UWB is widely used in indoor positioning due to its high transmission rate, strong anti-multipath interference capability, and high positioning accuracy. However, this indoor sensing capability of UWB is strongly influenced by environmental obstructions. Different occlusions—such as wooden doors, solid walls, or the human body—alter propagation paths and first-path energy, causing systematic ranging bias. Therefore, correcting NLOS errors is crucial for UWB positioning [11].

Generally speaking, research on NLOS error correction can be categorized into two types: (1) correcting errors directly without classifying sample data and (2) classifying the target first and then correcting based on the classification results. The method proposed in this study is based on correction according to classification results, and thus, we focus on introducing the second approach. Qi et al. [12] utilized Channel Impulse Response (CIR) of received UWB signals to classify channel conditions, and through Extended Kalman Filter (EKF) positioning and Long Short-Term Memory (LSTM) training of the observed channel state, the method alleviated the positioning degradation caused by NLOS conditions. Guo et al. [13] detected NLOS using a sliding window method, achieving 90% accuracy, and designed a delay model to reduce the error caused by UWB signals propagating through walls, which allows the method to achieve positioning accuracy close to LOS in NLOS environments. Kunze et al. [14] proposed an efficient NLOS recognition scheme based on a multi-input learning (MIL) neural network model, which uses CIR and CIR time-frequency diagrams (TFDOCIR), achieving an overall NLOS recognition accuracy of 91.74%. Deng et al. [15] represented the probability distribution of LOS signal samples using the mean and covariance of the first two matrices and then used the probability distribution and all NLOS signal samples to build the model. This approach does not rely on the number of LOS signals and is suitable for handling the imbalanced classification problem between LOS and NLOS signal samples, outperforming LS-SVM and SVDD. Deng et al. [16] proposed a TinyML solution assisted by an attention mechanism, which utilizes self-attention to enhance the effective representation ability of the pre-trained classifier model and accelerates inference efficiency at the edge using Post-Training Quantization (PTQ) strategy, surpassing all baseline strategies in PC-side performance. Currently, scholars worldwide combine CIR (Channel Impulse Response) with deep learning methods for NLOS error correction. However, channel feature analysis methods [17,18,19,20] still require users to perform secondary development of UWB hardware and software, and the computational complexity makes these methods unsuitable for low-cost UWB devices. The algorithms mentioned above combine CIR with deep learning, but they have issues such as excessive feature dimensions or limited identification of NLOS error sources.

In complex indoor environments, NLOS errors can cause large UWB positioning errors. This paper proposes a three-stage pipeline: classification, compensation, and positioning. We classify NLOS sources using received power and ranging features with a random forest model and a threshold rule based on the squared adjacent ranging difference. We then use a multi-layer perceptron (MLP) to learn a nonlinear compensation model for each obstruction type. We finally estimate positions with nonlinear least squares (NLS) using corrected ranges from double-sided two-way ranging (DS-TWR).

## 2. Materials and Methods

**Figure 1:**
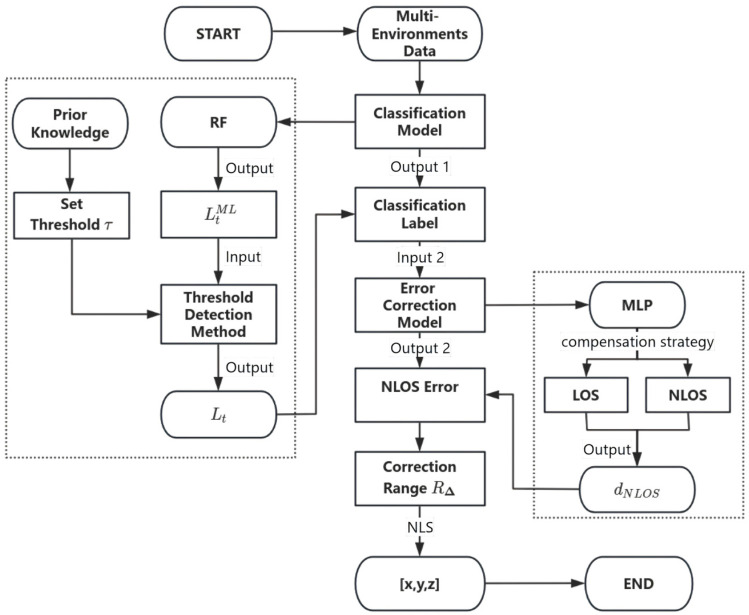
Flowchart for the proposed Recognition and Compensation Method for UWB Ranging Errors.

To improve the accuracy and stability of UWB positioning technology in complex indoor environments, this paper proposes a novel solution consisting of three main components: error identification, environment-adaptive correction, and positioning optimization. The process of the proposed method is illustrated in Figure 1.

### 2.1. UWB Low-Dimensional Feature Extraction

The UWB system uses the UML1 module from HaoRu Technology, developed on the DWM1000 platform, with the antenna delay set to 16,445 and employing Double-Sided Two-Way Ranging (DS-TWR) for distance measurement. After receiving the physical layer signals and analyzing the channel, it outputs multiple low-dimensional channel features, including total received power (TR) and first-path power (FP).

#### 2.1.1. Extraction of DS-TWR Ranging Measurements

During the DS-TWR ranging process, the tag first transmits a Poll frame to the base station. After receiving the Poll, the base station sends a Response frame after a reply delay. The tag then transmits a final frame. Four timing intervals are used in the ranging computation: $T_{\mathrm{round}1}$, the round-trip interval measured at the tag side from transmitting the Poll frame to receiving the Response frame; $T_{\mathrm{reply}1}$, the reply delay measured at the base-station side from receiving the Poll frame to transmitting the Response frame; $T_{\mathrm{round}2\;}$, the round-trip interval measured at the base-station side from transmitting the Response frame to receiving the Final frame; and $T_{\mathrm{reply}2}$, the reply delay measured at the tag side from receiving the Response frame to transmitting the Final frame. Based on these timing records, the DS-TWR range estimate used in this study is calculated as:

(1) $$R\;=c\cdot \frac{T_{\mathrm{round}1}T_{\mathrm{round}2}-T_{\mathrm{reply}1}T_{\mathrm{reply}2}}{T_{\mathrm{round}1}+T_{\mathrm{reply}1}+T_{\mathrm{round}2}+T_{\mathrm{reply}2}}$$

where c is the speed of light constant, and R is the estimated distance between the tag and the base station. Equation (1) gives the DS-TWR range estimate calculated from the recorded timestamps, rather than the true geometric distance itself.

In this study, the primary measurement is the UWB time-of-flight-based ranging observation obtained via the DS-TWR protocol. Under ideal line-of-sight conditions, the ranging error is dominated by random noise, whereas under NLOS conditions, additional systematic bias and increased uncertainty are introduced. Therefore, mitigating measurement-level errors is essential for improving the overall positioning performance.

#### 2.1.2. Extraction of Channel Features

The UWB module in this study performs signal reception and Channel Impulse Response (CIR) analysis at the hardware level, while the firmware calculates the total received power (TR) and first-path power (FP).

After completing each ranging cycle, the tag module encapsulates the timestamp, range measurements, and the corresponding TR/FP features into an uplink data message. The message is transmitted in real time through a serial port at 115,200 bps with an 8-N-1 format and is received by the XCOM serial debugging tool for real-time display and data logging. The recorded serial data are then used for subsequent experimental data organization and algorithm analysis. The data are sent in a fixed protocol format, with the message header “mc”, as shown in Table 1.

Consider anchor A0 and target T0 as an example. At timestamp i, the serial port transmits the following message:

(2) $$“\mathrm{mc},\;i,\;R_{i},\;{\mathrm{TR}}_{i},\;{\mathrm{FP}}_{i},\;\mathrm{END}”$$

where mc and END are the message header and trailer; i is the time; $R_{i}$ is the ranging value of anchor A0 to target T0 at this time; $TR_{i}$ is the total received power of the signal propagation from anchor A0 to target T0 at this time; ${\mathrm{FP}}_{i}$ is the received power of the first path.

In order to effectively distinguish between LOS and NLOS states, this paper defines the power difference as:

(3) $${\Delta P}_{i}\;={\;\mathrm{TR}}_{i}\;-\;{\mathrm{FP}}_{i}$$

where ΔP is the power difference.

Based on the ranging value ($R_{i}$) obtained by DS-TWR, a two-dimensional feature vector is constructed:

(4) $$F\;=\;\{R_{i},{\;\Delta P}_{i}\}$$

where F is the two-dimensional feature vector. It should be noted that this two-dimensional feature vector is only used for occlusion classification, whereas the subsequent compensation stage uses a separate three-dimensional input composed of R, FP, and TR.

Based on the DS-TWR ranging value R and the power-difference feature ΔP, a lightweight two-dimensional feature vector is constructed for the subsequent classification stage. The feature dimension is low, the computational cost is small, and the features can be output by the module in real time without additional CIR parsing and processing. In the experiments of this study, for the same anchor–tag link, the variation in the first-path power FP is relatively small, while NLOS effects are mainly reflected in the attenuation of the total received power TR. Therefore, the feature ΔP = TR − FP mainly captures the propagation-state-related power variation and is effective for distinguishing LOS and NLOS-like conditions in the considered scenarios.

### 2.2. Machine Learning-Based UWB NLOS Classification Model with Threshold Matching Assistance

**Figure 2:**
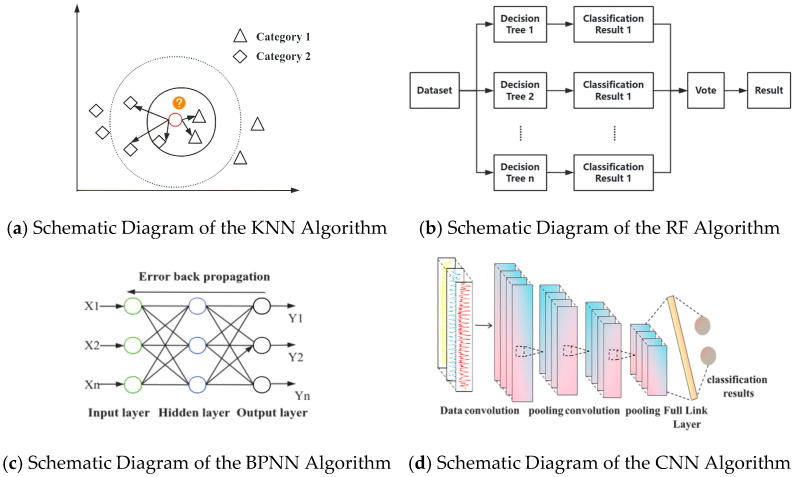
Representative candidate classifiers considered for low-dimensional UWB NLOS recognition.

Figure 2 illustrates the representative classifiers considered in this study, including KNN [21], RF [22], BP [23], and CNN [24], which were compared to determine the most suitable model for low-dimensional UWB NLOS recognition. Their comparative results are presented later in Section 3.2. The model that exhibits the best classification performance is selected for subsequent environmental recognition. Since the RF model achieved the best overall recognition performance and showed more stable behavior across different occlusion conditions, it was selected as the classifier in the proposed framework. This section focuses on the threshold-assisted temporal correction strategy built on top of the classifier output, rather than providing detailed introductions to standard classification algorithms. Accuracy was used as the performance evaluation metric. Since the task in this study is a four-class classification problem involving LOS, door, wall, and body occlusion, the classification accuracy is defined as:

(5) $$\mathrm{Acc}\;=\;N_{corr}/\;N_{total}$$

where Acc is the multi-class classification accuracy, $N_{corr\;}$ denotes the number of correctly classified samples, and $N_{total}$ denotes the total number of evaluated samples.

**Figure 3:**
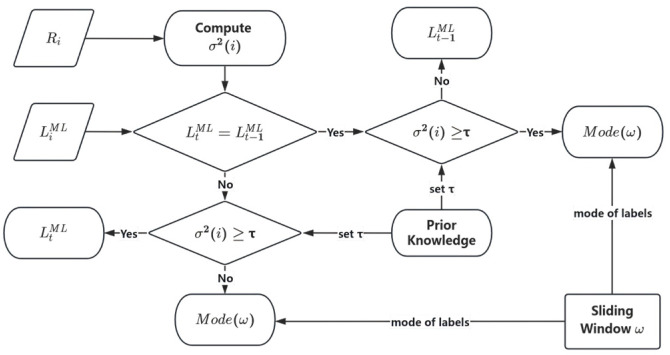
Flowchart of the Sliding Window-Based Threshold Detection and Correction Model.

In dynamic positioning, while machine learning models based on low-dimensional physical features can distinguish LOS from various NLOS states, their instantaneous outputs are prone to errors caused by CIR jitter, quantization noise, and short-term environmental fluctuations. These frame-level misclassifications disrupt label consistency and hinder subsequent error modeling and position estimation. This section introduces a threshold-based dynamic label correction method. It analyzes the statistical stability of squared adjacent ranging differences to identify anomalous jumps in the classifier’s output, enabling conditional correction of erroneous labels while preserving sensitivity to actual propagation changes. The specific structure is shown in Figure 3.

At a sampling frequency of 50 Hz, the interval between two consecutive range measurements is only 0.02 s. Let the pedestrian’s speed be v = 1.2 m/s [25], and the angle between the pedestrian’s movement direction and the line connecting the anchor and the target be θ. In this case, only the radial displacement contributes to the range measurement.

(6) $$\Delta d_{\mathrm{radial}}=v\Delta \mathrm{tcos}\theta$$

where $\Delta d_{\mathrm{radial}}$ is the radial displacement measurement, θ is the angle between the pedestrian’s movement direction and the line connecting the anchor and the target, and v is the pedestrian’s speed.

(7) $$\Delta d_{\mathrm{radial}}^{\;\max}=1.2\;\times \;0.02\;\times \;\cos0{}^{\circ}=0.024\;m$$

When the travel direction aligns perfectly with the base station-label line (θ = 0°), the maximum value of the adjacent ranging difference can be obtained.

(8) $$\Delta d_{\mathrm{radial}}^{\;\min}=1.2\;\times \;0.02\;\times \;\cos85{}^{\circ}=0.002\;m$$

(9) $$\sigma^{2}\left(t\right)={∆d}^{2}$$

The kinematic analysis demonstrates that adjacent ranging variations under normal pedestrian movement are only millimeter-scale. Building on this, we utilize the squared of adjacent ranging difference sequences as a metric for propagation stability. The squared of adjacent ranging difference $\sigma^{2}\left(t\right)$ reflects both small-scale motion disturbances and propagation-induced jumps. To distinguish the two, a physically meaningful threshold τ is required.

For penetration-type NLOS conditions, the occluding object is approximated as a homogeneous thin dielectric layer, and the signal is assumed to pass through the layer under near-normal incidence. In air, the electromagnetic wave propagation velocity is approximately c, while in a material with refractive index $r_{i}$, the propagation velocity can be approximated as $c/r_{i}$. Therefore, when the signal penetrates an occluding layer of thickness w, an additional propagation delay is introduced relative to propagation through the same thickness in air. Converting this delay into an equivalent excess path length yields a first-order approximation proportional to $w\left(r_{i}\;-1\right)$. Based on this approximation and the penetration-based NLOS analysis reported in [26], Equation (10) is used in this study to describe the additional NLOS ranging bias caused by weak penetration-type obstructions.

(10) $$d_{\mathrm{NLOS}}=\hat{d}\;-\;d\;=w\left(r_{i}\;-\;1\right)$$

where $d_{\mathrm{NLOS}}$ represents the NLOS error caused by the occluding object, $\hat{d}$ indicates the range given by UWB, d denotes the measured distance, w represents the total width of the obstructing wall, and $r_{i}$ denotes the refractive index of the occluding object.

Among the four obstruction types considered (LOS, wooden door, wall and human body), the wooden door produces the smallest excess path delay and therefore represents the mildest form of NLOS that could be mistaken for LOS.

From the equation above, it can be seen that the NLOS error is directly proportional to the dielectric constant and thickness of the occluding object. Therefore, the threshold for detecting environmental changes should be close to the minimum NLOS error caused when the signal passes vertically through a 4 cm wooden door. With a refractive index of 1.41 for the wooden door [26], the squared NLOS error is approximately 0.0003. This value represents the smallest effective disturbance separating LOS from the mildest NLOS condition. Considering non-normal incidence, a conservative threshold is selected as:

(11) $$\tau \;=\;0.0005{\;m}^{2}$$

where τ is the threshold.

This threshold is intended to separate millimeter-level motion-induced fluctuations from centimeter-level disturbances caused by propagation-state changes. It should be noted that τ is selected for the current experimental setting, namely a sampling frequency of 50 Hz and pedestrian-scale motion, and should therefore be interpreted as an application-oriented parameter rather than a universally fixed constant. If the target speed changes significantly, the adjacent ranging difference and its squared value may also increase accordingly. In such cases, the threshold should be retuned or adaptively adjusted according to the motion speed and sampling frequency. Since all experiments in this study were conducted under typical walking-speed conditions, the selected threshold was found to be appropriate and effective for the considered scenarios.

After determining the threshold, a dynamic correction approach is proposed by comparing the continuity of $L_{t}^{ML}$ and $L_{t-1}^{ML}$, as well as the magnitude relationship between τ and $\sigma^{2}\left(t\right)$. This approach is based on dynamic correction using sliding-window threshold detection.

To handle various correction scenarios, a sliding window $\omega_{t}$ of size 5 is defined as follows:

(12) $$\omega_{t}=\;\{R_{t-2},R_{t-1},R_{t},R_{t+1},R_{t+2}\}$$

where $R_{t}$ is the t-th ranging value, $R_{t-2}$ and $R_{t-1}$ are the two adjacent ranging values before the t-th ranging value, and ${\;R}_{t+1}$, $R_{t+2}$ are the two adjacent ranging values after the t-th ranging value.

When approaching the sequence boundary (e.g., t < 2), the window is automatically truncated based on available samples, with only the valid portion constituting $\omega_{t}$.

The final time consistency label is determined by majority voting, as expressed in the following formula:

(13) $$Mode\left(\omega_{t}\right)=arg\underset{c\in C}{max}\sum_{n\in \omega_{t}}I\left(L_{n}^{ML}=c\right)$$

where $Mode\left(\cdot \right)$ is the function for calculating the mode, $\omega_{t}$ denotes the sliding window constructed at time t, c∈R,0≤c≤3, and $L_{n}^{ML}$ represents the n-th label output by the machine learning model within the window. $I\left(L_{n}^{ML}=c\right)$ is the indicator function, used to count the occurrences of a specific category within the window. $arg\underset{c\in C}{max}$ denotes the category with the highest occurrence frequency among all candidate categories.

For time i, if the classification model outputs $L_{t}^{ML}=L_{t-1}^{ML}$, it indicates that the current label matches the previous one:

If $\sigma^{2}(i)<\tau$, the propagation state is assumed unchanged, and the final category label $L_{t}$ is set as:

(14) $$L_{t}=\;L_{t-1}^{ML}$$

If $\sigma^{2}(i)\geq \tau$, we assume a change in transmission status. However, due to CIR jitter or short-term environmental fluctuations causing model misjudgment, $L_{t}$ is set based on the mode of label types within the window.

(15) $$L_{t}=Mode\left(\omega_{t}\right)$$

where, $\omega_{t}$ denotes the sliding window constructed at time t, and $Mode\left(\cdot \right)$ is the function for calculating the mode.

For each time point t, if the error recognition model outputs $L_{t}^{ML}\neq L_{t-1}^{ML}$, then:

When $\sigma^{2}(i)<\tau$, the propagation remains stable, indicating no substantial structural changes in the current propagation environment over short time scales. In such cases, label jittering is more likely caused by CIR fluctuations, transient multipath enhancement, or measurement noise-induced misjudgments rather than actual LOS/NLOS state transitions. To mitigate label jitter and enhance temporal consistency under the short-term environmental stability assumption, the mode $\omega_{t}$ of labels within the sliding window $Mode\left(\omega_{t}\right)$ is adopted as a robust estimate, yielding the final category label $L_{t}$:

(16) $$L_{t}=\;Mode\left(\omega_{t}\right)$$

If $\sigma^{2}(i)\geq \tau$, the propagation path occlusion of the frame display signal changes, indicating environmental variation; then the error recognition model is considered to have classified correctly, no correction is performed, and the final category label $L_{t}$ is output:

(17) $$L_{t}=\;L_{t}^{ML}$$

The final category label $L_{t}$ is obtained by integrating the error recognition model assisted by threshold detection based on the sliding window method.

### 2.3. Environmental Adaptive Error Delay Correction Model

**Figure 4:**
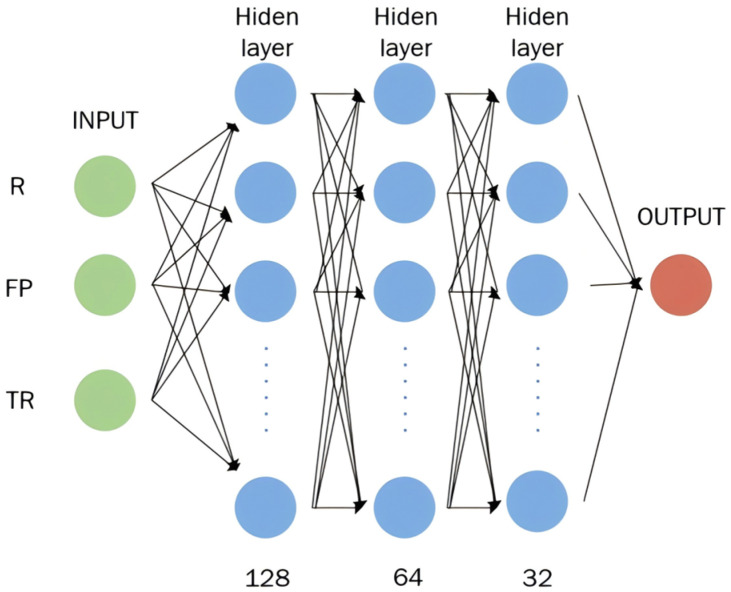
Multilayer Perceptron with three hidden layers.

UWB ranging errors exhibit significant variations under different occlusion environments. In LOS conditions, the error is relatively small and stable, while in NLOS conditions such as human occlusion or wall obstruction, the error distribution is complex and the magnitude increases significantly. Traditional models often apply a uniform error correction strategy, making it difficult to accurately model error distribution characteristics in different environments. To address this issue, this paper proposes an environmental adaptive error compensation model based on a Multilayer Perceptron (MLP) [27], which, in combination with the aforementioned classification results, performs environment-specific error prediction and correction. The model structure is shown in Figure 4.

The model uses the raw ranging values (R), the first-path received power (FP), and the total received power (TR) as the main inputs, which reflect the distance to the object, the direct signal strength, and the multipath attenuation level of the environment, respectively. The model uses the difference between predicted errors and actual errors as the loss function, and through multiple iterations, it minimizes the loss to improve the accuracy of the model in predicting error values in various indoor environments.All input features undergo Z-score normalization to eliminate the influence of dimensionality and enhance the model’s generalization ability.

(18) $$X_{\mathrm{scale}}=\frac{X\;-\;\mu }{\sigma }$$

where $X\;=\;{\left[R,\mathrm{FP},\mathrm{TR}\right]}^{T}$, μ is the mean of the training set, and σ is the standard deviation of the training set.

The network structure consists of three fully connected hidden layers, with the number of nodes being 128, 64, and 32, respectively. The ReLU activation function is used, and Batch Normalization and Dropout mechanisms are embedded between layers to accelerate convergence and prevent overfitting. The model ultimately outputs the error estimation value for each sample, which serves as the correction term to update the raw ranging value. Considering the significant differences in error statistical characteristics under different occlusion types, this paper trains separate MLP sub-models for four distinct environments: LOS, door, wall, and body. In practical applications, the classification model first labels the ranging samples with their environment, and then calls the corresponding sub-model to generate the error prediction value.

(19) $$\begin{array}{c} h_{1}=Dropout\left(\mathrm{ReLU}\left(\mathrm{BN}\left(W_{1}^{T}X\right)\right)\right)\\ h_{2}=Dropout\left(\mathrm{ReLU}\left(\mathrm{BN}\left(W_{2}^{T}h_{1}\right)\right)\right)\\ \Delta =d_{\mathrm{NLOS}}=W_{3}^{⊤}\left(\mathrm{ReLU}\left(W_{4}^{⊤}h_{2}\right)\right) \end{array}$$

where BN stands for Batch Normalization, which normalizes the output of each layer; ReLU is the activation function. $h_{1}$ represents the first layer fusion, $h_{2}$ represents the second layer fusion, and Δ represents the fusion output result.

(20) $$\Delta ={\;d}_{\mathrm{NLOS}}=f\left(R,\mathrm{FP},\mathrm{TR}|\mathrm{env}\right)\;,\;\mathrm{env}\in \;\left\{\mathrm{los},\mathrm{door},\mathrm{wall},\mathrm{body}\right\}$$

where $d_{\mathrm{NLOS}}$ is the NLOS error, and env represents the LOS/NLOS environment. f(·) is the functional relationship established by the machine learning model between the target result and the input features.

This design enables the error compensation process to have good environmental adaptability and nonlinear expression capability, avoiding fitting biases that may occur in a unified model under mixed environments. A dynamic learning rate decay strategy and early stopping mechanism are employed during training to further enhance the stability of the model and improve convergence efficiency.

### 2.4. Nonlinear Least Squares Positioning Model Based on DS-TWR Ranging

After error classification and range correction, the node position is estimated from the corrected DS-TWR observations using a nonlinear least squares (NLS) [28] formulation. This choice is motivated by the fact that the corrected measurements remain geometric range observations, and the corresponding positioning problem is naturally expressed as the minimization of nonlinear range residuals. In this study, NLS is adopted as the back-end estimation method because of its simplicity, effectiveness, and ease of implementation. It should also be noted that the proposed framework is not restricted to NLS; other back-end estimators, such as weighted least squares or robust filtering methods, could also be integrated if required.

By incorporating the error estimation values Δ from the previously mentioned MLP model, the ranging result $R_{i}$ is compensated to obtain the corrected distance:

(21) $${\hat{R}}_{i}\;=\;R_{i}\;-\;\Delta_{i}$$

where $\hat{R}$ is the corrected distance, $R_{i}$ is the ranging value, and Δ is the error estimation value.

Let there be n base stations, with known coordinates $\left(x_{i},y_{i}\right)$, and the unknown position of the node is (x,y). The geometric distance between the node and the i base station is:

where $d_{i}$ is the geometric distance between the node and base station.
  (22) $$d_{i}=\sqrt{(x-\;x_{i}{)}^{2}+(y\;-\;y_{i}{)}^{2}}$$

The ranging residual $e_{i}$ is defined as the difference between the corrected ranging value ${\hat{R}}_{i}$ and the geometric distance${\;d}_{i}$:

(23) $$e_{i}\;=\;{\hat{R}}_{i}\;-\;d_{i}$$

where $e_{i}$ is the ranging residual. By stacking the residuals from all base stations, the residual vector can be constructed as:

(24) $$e={\left[e_{1},\;e_{2},\;\ldots ,\;e_{n}\right]}^{T}$$

where e is the residual vector.

By constructing the residuals from all the base stations, the objective function is established as a nonlinear least squares problem that minimizes the sum of the squared residuals:

(25) $$\underset{x,y}{\min}\;\sum_{i=1}^{n}e_{i}^{2}=\;\underset{x,y}{\min}\;\sum_{i=1}^{n}\;{\left({\hat{R}}_{i}\;-\;\sqrt{(x\;-\;x_{i}{)}^{2}\;+\;(y-{\;y}_{i}{)}^{2}}\right)}^{2}$$

where $\sum_{i=1}^{n}e_{i}^{2}$ is the sum of squared residuals, $\underset{x,y}{\min()}$ is the minimum function.

Since the above objective function is nonlinear and cannot be solved analytically, the Levenberg–Marquardt algorithm is adopted for iterative optimization. At the k-th iteration, let the current position estimate be $\left(x^{k},y^{k}\right)$, and δ denote the position increment. By performing first-order linearization of the residual vector around the current estimate, one obtains:

(26) $$e(P^{\left(k\right)}+\delta )\approx eP^{\left(k\right)}+J\delta$$

where J is the Jacobian matrix of the residual vector with respect to the position variables, and $P^{\left(k\right)}$ is the current position estimate, and $P^{\left(k\right)}={\left[x^{k},y^{k}\right]}^{T}$. Then, the position increment satisfies:

(27) $$(J^{T}J\;+\;\lambda I)\delta \;=\;-J^{T}e$$

where λ is the damping factor, I is the identity matrix, and δ is position increment. The updated node position is:

(28) $$(x^{k+1},y^{k+1})\;=\;(x^{k},y^{k})\;+\;\delta$$

where $x^{k+1},\;y^{k+1}$ is the updated position estimate, $x^{k},\;y^{k}$ is the current estimate.

The iteration terminates when the residual reduction is smaller than a predefined threshold or when the maximum number of iterations is reached. Since iterative optimization is sensitive to the initial value, an anchor-centroid initialization is adopted in the experiments. For the current four-anchor convex layout, this initialization corresponds to the geometric center of the anchor configuration. For irregular, non-quadrilateral, or more complex anchor deployments, the initial position can be obtained from the centroid of all available anchors or from a coarse estimate, such as linear least squares or weighted least squares, and then refined using the same nonlinear optimization procedure. Therefore, the initialization strategy mainly affects convergence behavior and numerical robustness, but does not change the formulation of the proposed positioning framework.

With the incorporation of error compensation, this positioning model fully leverages the stability of DS-TWR ranging and the environmental correction ability of the MLP model, exhibiting excellent positioning accuracy and convergence in both static and dynamic scenarios. The next chapter will further validate the model’s comprehensive positioning performance under different occlusion environments through experiments.

## 3. Results

This section evaluates and verifies the proposed method from two perspectives: static scenarios and dynamic paths. The experiments aim to analyze the classification model’s recognition accuracy, the error compensation model’s correction ability, and the overall improvement in positioning accuracy. All experiments were conducted on a UWB ranging system based on the DS-TWR protocol, with four typical occlusion conditions in a real indoor environment selected for testing, including LOS, door occlusion (door), wall occlusion (wall), and body occlusion (body).

### 3.1. UWB Channel Features Validation

**Figure 5:**
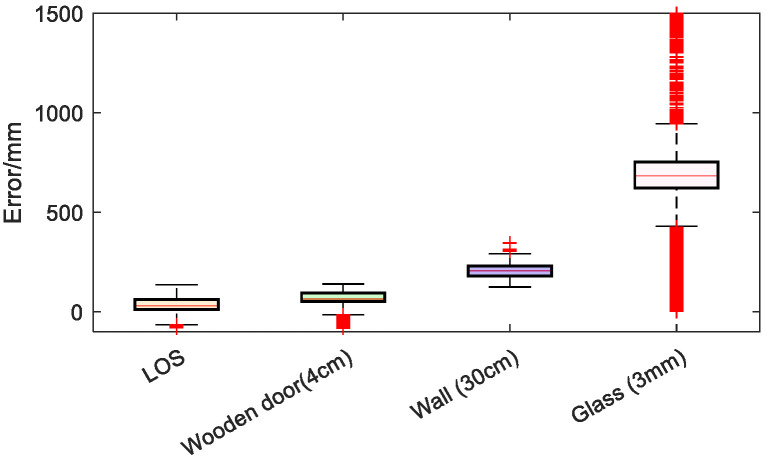
Boxplot of UWB ranging errors under different occlusion conditions.

**Figure 6:**
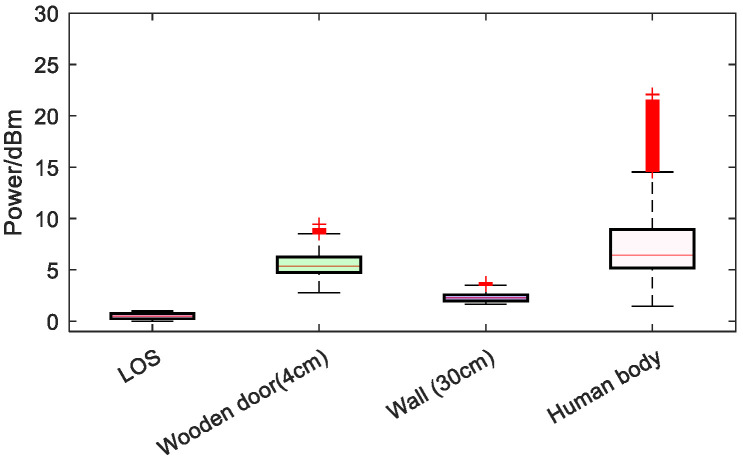
Boxplot of the difference between the total received power and the first path power in LOS and NLOS environments.

To further validate the discriminative capability of low-dimensional channel features across diverse environments, this study analyzed the statistical distribution of ranging error and received power difference characteristics under four typical propagation scenarios: line-of-sight (LOS), 4 cm wooden door obstruction, 30 cm wall obstruction, and human body obstruction. Figure 5 presents the box plot of ranging error, while Figure 6 displays the box plot of received power difference.

Figure 5 shows that the error is smallest and most concentrated in the LOS scenario. In the wooden-door and wall-occlusion scenarios, the error becomes larger and its distribution becomes wider. In the human-body-occlusion scenario, the error is the largest and the most dispersed, indicating obvious non-line-of-sight characteristics. Figure 6 demonstrates a high consistency between the received power difference characteristics and the error trend. In the line-of-sight (LOS) scenario, ΔP approaches zero and remains stable, indicating that the first-order energy is consistent with the total energy. The ΔP significantly increases due to obstructions from wooden doors and walls, reflecting a higher proportion of multipath energy. Under human occlusion conditions, ΔP reaches its maximum with significantly enhanced fluctuation amplitude, indicating further attenuation of first-order energy, while backscattering and non-first-order components dominate.

This validation demonstrates that the power-difference feature ΔP exhibits a distinct monotonic response to occlusion intensity while maintaining good separability across diverse scenarios, making it an effective environment-sensitive feature for the classification stage.

The results show that the low-dimensional channel energy difference feature proposed in this study has good physical interpretability and practical effectiveness, which provides a solid foundation for the subsequent model training and performance improvement.

### 3.2. Data Collection and Model Training

**Figure 7:**
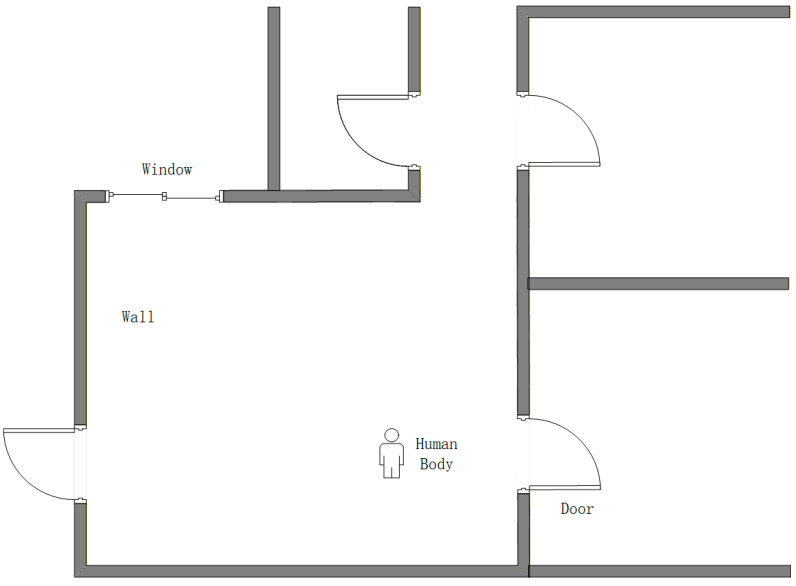
Experimental scene of the indoor environment.

To achieve accurate occlusion environment recognition, four classification models were trained on the preprocessed UWB ranging data: KNN, BP, CNN, and RF. The model training used static data, covering four typical occlusion environments: LOS, door, wall, and body. Data collection environments are shown in Figure 7. Approximately 4200 ranging samples were collected for each environment, and the training and testing datasets were divided at an 80:20 ratio. For classification, the input feature vector was defined as [R,∆P], where R is the raw ranging value and ∆P=TR−FP is the power-difference feature. To eliminate the influence of dimensionality, the features were standardized using Z-score normalization before model training.

Table 2 presents a comparative analysis of classification accuracy across four models under four distinct environmental conditions. The results indicate that the RF model demonstrates robust generalization capabilities across all environments, achieving an average accuracy exceeding 88%. The KNN model ranks second, while also exhibiting relatively consistent recognition performance. In contrast, both the CNN and BP models exhibit insufficient stability in scenarios with severe occlusion, where their accuracy rates fall below 70%.

The environmental labels generated by the classification models serve as the input basis for the error compensation models. To address each specific occlusion environment, a dedicated MLP sub-model was trained, resulting in a total of four distinct compensation models. These models utilize ranging values and received power features as inputs to predict the error compensation value ∆. Each MLP model incorporates three hidden layers (128, 64, and 32 neurons), utilizes activation functions, and employs the MSE loss function.

**Figure 8:**
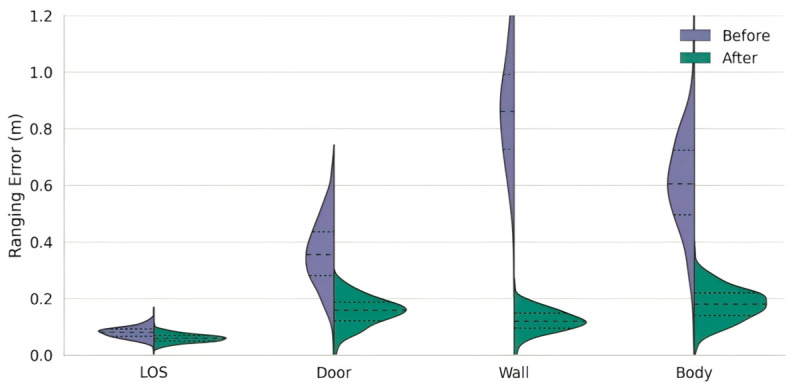
Violin plots of ranging error distributions before and after error compensation under four occlusion environments.

Figure 8 illustrates the error compensation effects of the MLP model under four different environments. This figure shows violin plots before and after error compensation, where the blue represents the original ranging error, and the green represents the results after MLP correction.

In the LOS environment, the original error mean is 0.08 m, which slightly decreases to 0.06 m after compensation, with a slight improvement in the distribution convergence. In the door environment, the error mean decreases from 0.35 m to 0.15 m, achieving a compensation rate of 57%. In the wall scenario, the pre-compensation error mean is as high as 0.85 m, which drops to 0.12 m after correction, with an error reduction of over 85%. In the body occlusion environment, the error mean decreases from 0.61 m to 0.18 m, demonstrating good correction ability.

The violin plots also show the trend of error density concentration: before compensation, there is a right-skewed long-tail phenomenon, while after compensation, the median stabilizes, and the tail significantly contracts, with errors primarily concentrated within 0.2 m.

These results verify that the MLP model has strong nonlinear error modeling capabilities across different environments, especially performing exceptionally well in strong NLOS scenarios.

### 3.3. Experiments and Results Analysis in Static Scenario

To verify the effectiveness of the proposed error recognition and correction method in practical applications, static experiments were designed for comparative analysis. The experiment was conducted in a typical residential indoor environment. Four sample points, forming a square layout in a public area, were selected and labeled as points a, b, c, and d. Four UWB base stations are fixedly deployed at positions with different occlusion types (as shown in Table 3).

In this experiment, the ranging data is first classified, followed by error compensation using the MLP model corresponding to each environment. Finally, the position calculation is completed using the Nonlinear Least Squares (NLS) method. By comparing the corrected and uncorrected positioning results with the true coordinates, the Mean Absolute Error (MAE) and Root Mean Square Error (RMSE) are calculated to evaluate the error correction performance.

Taking point a as an example, Table 4 shows the classification results of the sample data using the Random Forest model. The recognition accuracy for LOS and NLOS light occlusion (wooden door) environments is the highest, reaching over 99%, and the classification accuracy for NLOS heavy occlusion areas is also around 85%.

**Figure 9:**
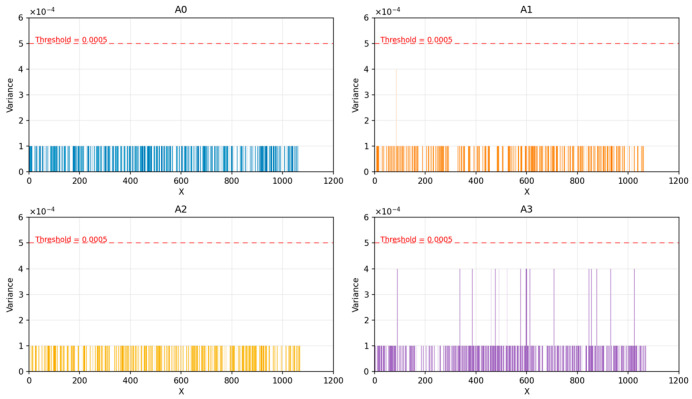
Comparison of the Squared Adjacent Ranging Difference with the Threshold.

With the assistance of the threshold detection method, the squared adjacent ranging difference for each station’s classification error samples and their adjacent time samples is calculated and compared with the threshold. As shown in Figure 9, the squared adjacent ranging difference for all four base stations during measurement is below the threshold, indicating that the signal propagation environment between the four base stations and the tag has not changed. Therefore, the incorrect category of the misclassified samples is corrected to the correct category.

**Figure 10:**
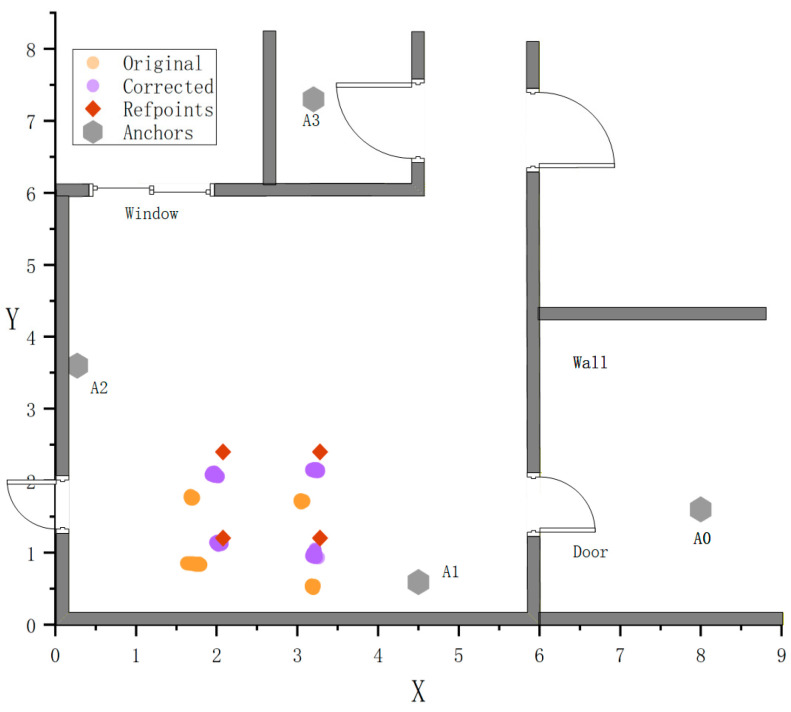
Comparison of Positioning Results Before and After Correction for Each Sample Point.

Figure 10 illustrates the relative positional relationships between the sampled data points and their true coordinates before and after correction for each sample. Table 5 shows that point b exhibits the most significant correction effect, with MAE and RMSE reduced by about 80%. Across all four sample points, the proposed method achieves substantial error suppression, yielding an average improvement of 65.16% in MAE and 65.12% in RMSE. These results fully validate the effectiveness of the proposed compensation strategy under static occlusion conditions.

(29) $$\mathrm{MAE}=\frac{1}{N}\sum_{n}\left|e_{\mathrm{corr}}^{\left(n\right)}\right|$$

where MAE is the mean absolute error, N is the total number of samples, n is the sample index, $e_{\mathrm{corr}}^{\left(n\right)}$ is the corrected error of sample n.

(30) $$\mathrm{RMSE}=\sqrt{\frac{1}{N}\sum_{n}{\left(e_{\mathrm{corr}}^{\left(n\right)}\right)}^{2}}$$

where RMSE is the square root of the mean squared corrected error.

Analyzing the spatial distribution of error reduction, it is evident that human occlusion and wall occlusion paths had the most significant impact on ranging before correction, leading to systematic shifts in the positioning results. However, by modeling occlusion recognition and environment-specific error compensation, such nonlinear errors were effectively suppressed, resulting in more stable corrected paths. This result indicates that the proposed method is highly adaptable and accurate in static environments, providing a reliable foundation for further application in dynamic scenarios.

It should be noted that the 100% classification accuracy achieved after threshold-assisted correction in the static scenario does not imply perfect intrinsic classification performance. In the static experiment, the occlusion condition remained unchanged over time. Therefore, the sliding-window threshold rule primarily acts as a temporal consistency enforcement mechanism, smoothing occasional misclassifications caused by transient measurement noise. The improvement results from eliminating isolated label fluctuations rather than fundamentally altering the classifier’s decision boundaries.

### 3.4. Experiments and Results Analysis in Dynamic Scenarios

To evaluate the dynamic adaptability of the proposed method in real complex indoor environments, a set of dynamic path experiments was designed. The experimental setup involved moving a tripod carrying a UWB tag along a predefined trajectory. The path contained multiple Non-Line-of-Sight (NLOS) regions and different types of obstructions, with the occlusion status changing in real-time as the person moved, simulating a dynamically complex propagation environment where the environmental label was unknown.

The ground truth trajectory was obtained by measuring predefined waypoints using a calibrated measuring tape and marking reference points on the floor. The coordinate system was established based on the known anchor positions, and all reference points were expressed in the same coordinate frame. During the dynamic experiment, the test subject followed the predefined path at a constant walking speed. Positioning errors were computed as the Euclidean distance between the estimated coordinates and the corresponding ground truth points.

The synchronization error between ground truth acquisition and UWB measurements was negligible compared with the positioning error magnitude.

To compare the effectiveness of the proposed method, three experimental schemes were set up as shown in Table 6:

- Scheme A serves as the baseline, relying solely on the original ranging values for positioning.
- Scheme B introduces classification and error compensation but does not use environmental consistency detection.
- Scheme C represents the proposed method, integrating classification, threshold detection, and compensation in a three-stage process.

**Table 6:** Comparative Setup of Three Schemes for Dynamic Positioning Tests.

	Scheme A	Scheme B	Scheme C
Classification Model	N/A	RF	RF + Threshold Detection
Error Model	N/A	MLP	MLP
Fitting Method	NLS	NLS	NLS

**Figure 11:**
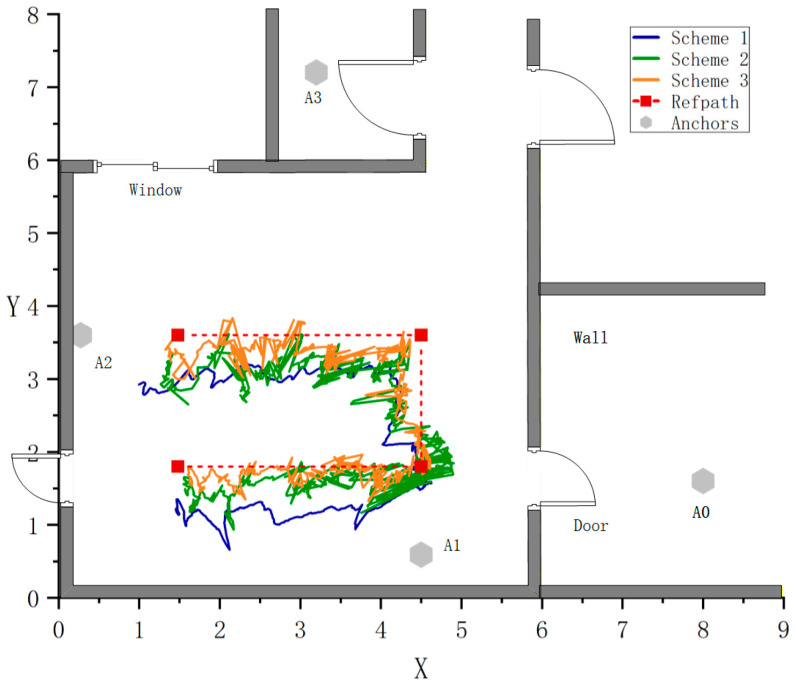
Comparison of Positioning Trajectories under Three Experimental Schemes.

Figure 11 shows the comparison of positioning trajectories and the predefined path for the three schemes. Scheme A’s fitted trajectory significantly deviates from the true path, especially at the corners, indicating that the original ranging errors have a significant impact on positioning. Scheme B’s trajectory generally follows the true path but still shows some deviation in areas with sudden changes in occlusion. Scheme C’s trajectory is the closest to the true path, with a smooth and continuous overall trajectory, clearly outperforming the other two schemes, demonstrating the synergistic effect of the environment recognition and compensation mechanisms.

**Figure 12:**
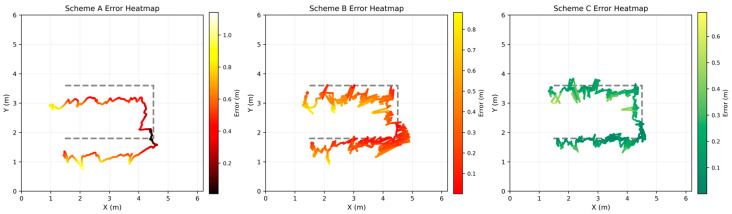
Heatmap of Trajectory Errors Under Different Schemes.

To more intuitively reflect the error distribution, Figure 12 presents the positioning error heatmaps for the three schemes. The color intensity, from dark to light, indicates the increase in error magnitude. In Scheme A, there are large areas with high errors. Scheme B shows slight improvement, while in Scheme C, the error region is significantly reduced, and the heatmap distribution is concentrated in the low-error region, indicating a significant improvement in positioning stability.

Table 7 summarizes the comparison of positioning accuracy for the three schemes in dynamic paths, including the MAE and RMSE metrics.

(31) $${\;\mathrm{Improvement}}_{\mathrm{MAE}}\left(\%\right)=\frac{{\mathrm{MAE}}_{\mathrm{orig}}\;-\;{\mathrm{MAE}}_{\mathrm{corr}}}{{\mathrm{MAE}}_{\mathrm{orig}}}\;\times \;100$$

where ${\;\mathrm{Improvement}}_{\mathrm{MAE}}\left(\%\right)$ is the percentage improvement in MAE, ${\mathrm{MAE}}_{\mathrm{orig}}$ is the MAE of the original (uncorrected) method and ${\mathrm{MAE}}_{corr}$ is the MAE after correction.

(32) $${\;\mathrm{Improvement}}_{\mathrm{RMSE}}\left(\%\right)=\frac{{\mathrm{RMSE}}_{\mathrm{orig}}\;-\;{\mathrm{RMSE}}_{\mathrm{corr}}}{{\mathrm{RMSE}}_{\mathrm{orig}}}\;\times \;100$$

where ${\;\mathrm{Improvement}}_{RMSE}\left(\%\right)$ is the percentage improvement in RMSE.

The results show that Scheme B achieves a decrease of 32.50% in MAE and 28.09% in RMSE compared to the original method (Scheme A). In contrast, the final Scheme C outperforms Scheme A, with an average decrease of over 60% in both metrics, significantly outperforming Scheme B. This highlights the importance of threshold detection in improving recognition consistency in dynamic environments.

Based on the above, the proposed method demonstrates stable environmental recognition and error correction capabilities in dynamic and variable occlusion scenarios. It exhibits good generalization ability and engineering usability, confirming its promising application prospects in real-world UWB deployments.

## 4. Discussion

This study proposes a lightweight recognition and compensation framework to mitigate UWB ranging errors in complex indoor environments. The experimental results demonstrate that the integration of NLOS classification, temporal consistency correction, and environment-adaptive error modeling significantly improves both ranging reliability and positioning accuracy under static and dynamic conditions.

The performance improvement primarily arises from two aspects. First, the sliding-window threshold mechanism enhances temporal consistency in classification results, effectively suppressing label jitter caused by transient multipath fluctuations and measurement noise. This prevents misclassification-induced compensation errors from propagating into the positioning stage. Second, the environment-specific MLP models capture nonlinear bias characteristics associated with different occlusion types. Compared with a unified correction strategy, the proposed environment-adaptive modeling approach better accommodates heterogeneous propagation conditions, especially in severe NLOS scenarios such as wall and human body occlusion.

Compared with CIR-based deep feature extraction methods, the proposed framework relies only on low-dimensional physical features directly available from commercial UWB modules. This avoids firmware-level modification and high-dimensional signal processing, thereby reducing computational complexity and improving deployability on low-cost devices. The results indicate that reliable NLOS mitigation can be achieved without accessing raw channel impulse response data.

Nevertheless, several limitations remain. The threshold selection strategy is derived under typical pedestrian motion assumptions and may require further adaptation in high-speed or highly dynamic environments. In addition, the experiments were conducted in representative indoor scenarios with predefined occlusion types; performance under more complex industrial or metallic environments warrants further investigation. Future work will explore adaptive threshold tuning and integration with additional sensors, such as inertial or vision-based systems, to further enhance robustness in challenging conditions.

## 5. Conclusions

This paper presented a lightweight three-stage framework for mitigating UWB ranging errors in complex indoor environments. By combining low-dimensional feature-based NLOS classification, sliding-window temporal consistency correction, and environment-specific MLP compensation with nonlinear least squares positioning, the proposed method significantly improves positioning accuracy in both static and dynamic scenarios.

The framework demonstrates that effective NLOS mitigation can be achieved without high-dimensional CIR feature extraction or hardware modification, making it suitable for practical deployment on commercial UWB devices. The proposed approach provides a scalable and engineering-friendly solution for enhancing indoor localization reliability. Future research will focus on adaptive parameter optimization and multi-sensor fusion to further improve performance in highly dynamic environments.

## Figures and Tables

**Table 1:** Serial Communication Protocol.

Content	Example	Function
HEAD	mc	Message header, fixed to mc
RANGTIME	100	Internal system timestamp of the MCU, ms
RANGE0	1.34	Distance to base station A0, meters
RANGE1	1.34	Distance to base station A1
RANGE2	1.34	Distance to base station A2
RANGE3	1.34	Distance to base station A3
A0 TR PWR	−88.94	Total received power of A0, dBm
A1 TR PWR	−88.94	Total received power of A1
A2 TR PWR	−88.94	Total received power of A2
A3 TR PWR	−88.94	Total received power of A3
A0 FP PWR	−88.94	First path power of A0, dBm
A1 FP PWR	−88.94	First path power of A1
A2 FP PWR	−88.94	First path power of A2
A3 FP PWR	−88.94	First path power of A3

**Table 2:** Statistical Accuracy of NLOS Error Recognition Based on Machine and Deep Learning Algorithms.

	RF	KNN	BP	CNN
LOS	87.26%	76.07%	61.38%	49.38%
Door	88.76%	79.47%	65.08%	50.63%
Wall	88.53%	79.63%	63.49%	48.32%
Body	88.45%	77.89%	63.02%	50.00%

**Table 3:** Base Station Occlusion Environments in Static Scenario.

	Point a	Point b	Point c	Point d
A0	Door	Door	Door	Door
A1	Body	Body	LOS	LOS
A2	LOS	LOS	Body	Body
A3	Wall	Wall	Wall	Wall

**Table 4:** Classification Performance of Each Anchor at Sampling Point a.

	A0	A1	A2	A3
True Category	Door	Body	LOS	Wall
Correct Sample Count	600	525	594	506
Accuracy	100%	87.51%	99.16%	84.35%
Accuracy After Threshold Detection Assistance	100%	100%	100%	100%

**Table 5:** Comparison of Positioning Accuracy for Sample Points Before and After Correction.

Point	MAE Before	RMSE Before	MAE After	RMSE After
a	0.7376	0.7377	0.3376	0.3377
b	0.4818	0.4824	0.0978	0.0982
c	0.6719	0.6720	0.2447	0.2455
d	0.7163	0.7163	0.2640	0.2641

**Table 7:** Comparison of Positioning Accuracy for Different Schemes.

Scheme	Scheme A	Scheme B	Scheme C
MAE	0.4968	0.3353	0.1597
RMSE	0.5383	0.3871	0.2023
MAE Improvement Percentage	\	32.51%	67.85%
RMSE Improvement Percentage	\	28.09%	62.42%

## Data Availability

The data presented in this study are openly available in Figshare, Available online: https://doi.org/10.6084/m9.figshare.31423238 (accessed on 2 February 2026).
